# Innovative reference materials for method validation in microplastic analysis including interlaboratory comparison exercises

**DOI:** 10.1007/s00216-023-04636-4

**Published:** 2023-03-22

**Authors:** Elena Martínez-Francés, Bert van Bavel, Rachel Hurley, Luca Nizzetto, Svetlana Pakhomova, Nina T. Buenaventura, Cecilie Singdahl-Larsen, Marie-Louise Tambo Magni, Jon Eigill Johansen, Amy Lusher

**Affiliations:** 1 Norwegian Institute for Water Research (NIVA), Økernveien 94, NO-0579 Oslo, Norway; 2 RECETOX, Masarik University, Kamenice 753/5, 625 00 Brno, Czech Republic; 3 Chiron AS, Stiklestadveien 1, 7041 Trondheim, Norway

**Keywords:** Microplastic reference material, Comparability, Comparison, Interlaboratory comparison study, Soda tablets, Soda capsules

## Abstract

Reference materials (RMs) are vital tools in the validation of methods used to detect environmental pollutants. Microplastics, a relatively new environmental pollutant, require a variety of complex approaches to address their presence in environmental samples. Both interlaboratory comparison (ILC) studies and RMs are essential to support the validation of methods used in microplastic analysis. Presented here are results of quality assurance and quality control (QA/QC) performed on two types of candidate microplastic RMs: dissolvable gelatin capsules and soda tablets. These RMs have been used to support numerous international ILC studies in recent years (2019–2022). Dissolvable capsules containing polyethylene terephthalate (PET), polyvinyl chloride (PVC), polyethylene (PE), and polystyrene (PS), in different size fractions from 50 to 1000 µm, were produced for one ILC study, obtaining relative standard deviation (RSD) from 0 to 24%. The larger size fraction allowed for manual addition of particles to the capsules, yielding 0% error and 100% recovery during QA/QC. Dissolvable capsules were replaced by soda tablets in subsequent ILC studies and recovery test exercises because they were found to be a more reliable carrier for microplastic RMs. Batches of soda tablets were produced containing different single and multiple polymer mixtures, i.e., PE, PET, PS, PVC, polypropylene (PP), and polycarbonate (PC), with RSD ranging from 8 to 21%. Lastly, soda tablets consisting of a mixture of PE, PVC, and PS (125–355 µm) were produced and used for recovery testing during pretreatment of environmental samples. These had an RSD of 9%. Results showed that soda tablets and capsules containing microplastics >50 µm could be produced with sufficient precision for internal recovery tests and external ILC studies. Further work is required to optimize this method for smaller microplastics (< 50 µm) because variation was found to be too large during QA/QC. Nevertheless, this approach represents a valuable solution addressing many of the challenges associated with validating microplastic analytical methods.

**Supplementary Information:**

The online version contains supplementary material available at 10.1007/s00216-023-04636-4.

## Introduction

Contamination of the environment with microplastics has gained significant attention in recent years. Microplastics, either generated through breakdown of larger plastic items or specifically produced to be small in size, are now recognized as a global problem [1, 2]. Microplastics present a risk to the environment and human health via contamination of water, food, soil, and air [2, 3]. They have been found in all environmental compartments from the atmosphere to the deep sea — in water, soil, sediments, and biota. Biota are exposed directly to microplastics through feeding or trophic transfer due to direct consumption and accumulation of plastic particles from consumed prey items [4–7]. Researchers therefore need to understand the consequences that microplastics will have on the environment, and have been working towards the development and optimization of methods and reporting protocols used for assessment in recent years [8–11].

Microplastics are a diverse suite of polymers, shapes, sizes, and additives [12] and thus a diverse and persistent pollutant [13]. Analytical methods and techniques available to identify and quantify microplastics in environmental matrices are numerous, covering many different polymers and size fractions. Widely validated and standardized methods are currently lacking [14, 15], and where similar methods are applied, there is often inconsistency between laboratories in how they report data [9, 16]. Currently, there are some tools available to help and support researchers ensure they are reporting enough details to guarantee high-quality outputs and produce comparable data and harmonization [9]. This includes sampling strategy, collection and preparation, analysis, quality assurance and quality control (QA/QC) criteria, and data management protocols [17], (www.EUROqCHARM.eu). The aim of these tools is to provide cross-comparable data that can be validated. One approach to compare methods used by different laboratories, or researchers, is method validation, whereby the reproducibility of a method can be assessed to return known, or spiked, reference material.

Nowadays, there is a lack of reference materials (RMs) for microplastic assessments to validate analytical methods. RMs are crucial for identifying appropriateness of methods, especially when accurate estimation or underestimation may occur for microplastics in environmental samples [16]. To date, there have been several attempts to generate RMs for microplastic studies. These usually take the form of including a known number of plastic particles into a sample and counting their recovery. This method has limitations as particles are often more uniform in shape (beads or pellets) and larger (> 1 mm) than microplastics typically found in environmental samples. Furthermore, validation of RMs is often lacking. More recently, researchers have been exploring cryo-milling and sonification to achieve more environmentally relevant particle shapes and size ranges in recovery tests [2, 18]. These have been applied to studies working with multiple laboratory methods and analytical approaches, including Fourier transform infrared (FTIR) spectroscopy and pyrolysis–gas chromatography–mass spectrometry (pyr-GCMS). Accurately validating RMs is necessary before their inclusion in microplastic studies. This can be done by controlling purity of produced RMs using approaches such as pyr-GCMS, Raman microscopy, or µFTIR analyses.

When performing spiking experiments and recovery tests, there are challenges associated with transporting and accurately dosing numbers of microplastic particles in different matrices. This is due to their small size and low weight, which can introduce errors. Moreover, manually counting small particles is time consuming, has potential for human error, and increases in difficulty with smaller particle sizes. Microplastics might also be influenced by static or adhere to vessels or containers preventing them from entering the sample/medium during spiking. An approach that encapsulates microplastics in a way that is sufficiently precise and can be added directly into the target media is needed. After proof-of-concept and suitability have been proven, the ultimate aim would be to produce certified RMs of several types and size fractions that have gone through a validation and certification process following the guidelines outlined in ISO 17034.

The aim of this work was to design, develop, and test easy-to-use candidate RMs (hereafter, RMs) for microplastic analysis. We present the development of a new concept with two different forms of RMs using soda tablets and soda capsules as a convenient way to include known amounts of microplastics into environmental samples. We include results of several validation tests performed on RMs, which were produced for different interlaboratory comparison (ILC) studies and in-house recovery tests. RMs consisted of single or multiple polymer mixes in size ranges from 50 µm to 1 mm for the most widely produced plastics: polypropylene (PP), polyethylene (PE), polystyrene (PS), polyethylene terephthalate (PET), polyvinyl chloride (PVC), and polycarbonate (PC).

## Materials and methods

**Fig. 1:**
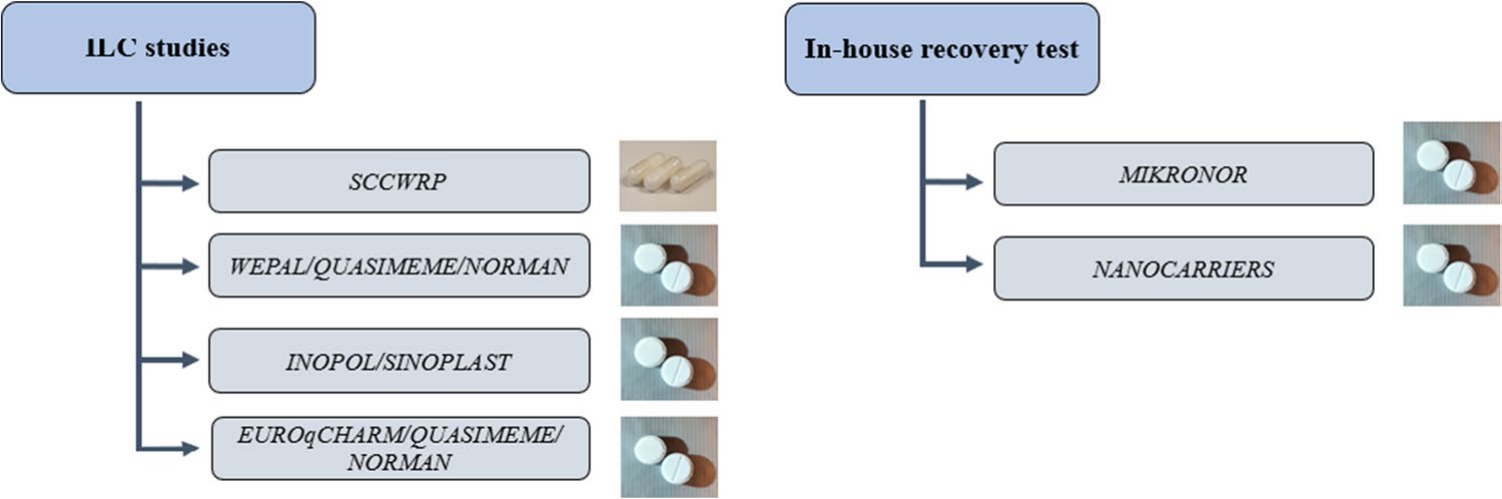
Summary of interlaboratory comparison (ILC) studies and in-house recovery tests using candidate reference materials (RMs) in the form of soda tablets and capsules

Microplastic RMs were prepared as soda tablets and capsules. All information regarding production and polymer types is explained in this section. A summary of the ILCs and recovery tests where the RMs were used is presented in Fig. 1. Specifics regarding the different polymer types and size fractions added to the soda tablets and capsules are presented in Table 1.

**Table 1:** Microplastics used in the production of candidate microplastic reference materials (RMs). The table displays the polymer types and size fractions included in RMs

Polymer type	Plastic type	Obtained from	Size fractions (µm)	Shape
PS	Powder	Goodfellow	50–100, 100–150, 150–355, and 425–500	Micro-fragments
PVC	Powder	Goodfellow	50–100, 150–250, and 250–350	Micro-fragments
PE	Powder	Cospheric	125–150, 425–500, and 850–1000	Microspheres
Blue PS	Powder	Unknown	1000	Micro-fragments
PET	Powder	Goodfellow	50–150 and 250–355	Micro-fragments
PET fibers	Fibers	IKEA	101–2194 length	Fibers
PE	Pellets	INEOS Norway	50–300	Micro-fragments
PET	Pellets	Sigma-Aldrich	50–300	Micro-fragments
PS	Pellets	Sigma-Aldrich	50–300	Micro-fragments
PC	Pellets	Goodfellow	50–300	Micro-fragments
PP	Pellets	Sigma-Aldrich	50–300	Micro-fragments
PVC	Pellets	INEOS Sweden	50–300	Micro-fragments

### Preparation of microplastics

Microplastics used in the production of RMs were sourced from commercially available material, purchased as powder from Goodfellow, UK, and Cospheric, USA, or by cryo-milling plastic pellets from Goodfellow, UK; Sigma-Aldrich, Norway; and INEOS, Norway and Sweden (Table 1). All microplastics in powder form purchased from Goodfellow were a mixture of different size fractions, so sieving of each polymer was required to separate them into size categories. Polymers in powder purchased from Cospheric were already size fractionated and were not sieved.

Cryo-milling was carried out for the later ILC study in collaboration with Chiron AS (Trondheim, Norway) using a SPEX® SamplePre Freezer/Mill®, Model 6875 D. This allowed inclusion of different microplastic size fractions to be generated from preproduction plastic pellets (Table 1). Cryo-milling was introduced to prepare microplastics which would be more similar to those found in environmental samples.

Microplastic RMs containing PET fibers were also made and were obtained by washing polyester blankets (“Skogsklocka”, IKEA, Norway) in a Candy smart washing machine “CS 1272D3/1-S” on a 15-min cycle program at 40 °C and centrifuging at 1200 rpm. The newly purchased washing machine was cleaned by running three washes on the same cycle as the blankets. No detergents or softeners were added. The effluent was then collected in a stainless steel pressure vessel and vacuum filtered through a 10-μm nylon membrane, which yielded fibers of 101–2194 μm in length and 29 μm in width.

### Preparation of microplastic RMs

#### Soda capsules

Dissolvable gelatin capsules produced for medical use were purchased from Capsule Connection, USA, and also kindly provided by Kragerø Tablet Production, AS, Norway. They were filled with a mixture of sodium hydrogen carbonate (NaHCO_3_) and malic acid (C_4_H_6_O_5_), provided by Kragerø Tablet Production, AS, Norway. These ingredients are stable when mixed in powder form but with the addition of a small amount of water they will effervesce. The mixture of ingredients is stoichiometrically balanced so that the end products are water and carbon dioxide, releasing particles into the medium in which they are added. Single polymer mixtures were prepared for PET, PE, PVC, and PS with different size fractions (from 3 µm to 1 mm) by weighing in known amounts of each polymer and combining thoroughly with pre-mixed powder formulation. This powder mixture was then transferred into capsules. During this process, a purpose-built tray housed one half of the capsule that was filled completely with powder mixture and closed with the empty second half of the capsule casing (Supplementary material, Fig. S1). The powder mixture and microplastic particles were mixed thoroughly using a shaker (300 rpm; 1 h) prior to filling the capsules. Size fractions produced below 50 µm were regarded as experimental based on limited research and development that had occurred in the research field and challenges associated with weighing and handling very small particles. Each combination of sizes and polymers was produced in batches of a 100, totaling 600 capsules per polymer and size fraction. Overall, 7200 soda capsules were produced.

#### Soda tablets

**Fig. 2:**
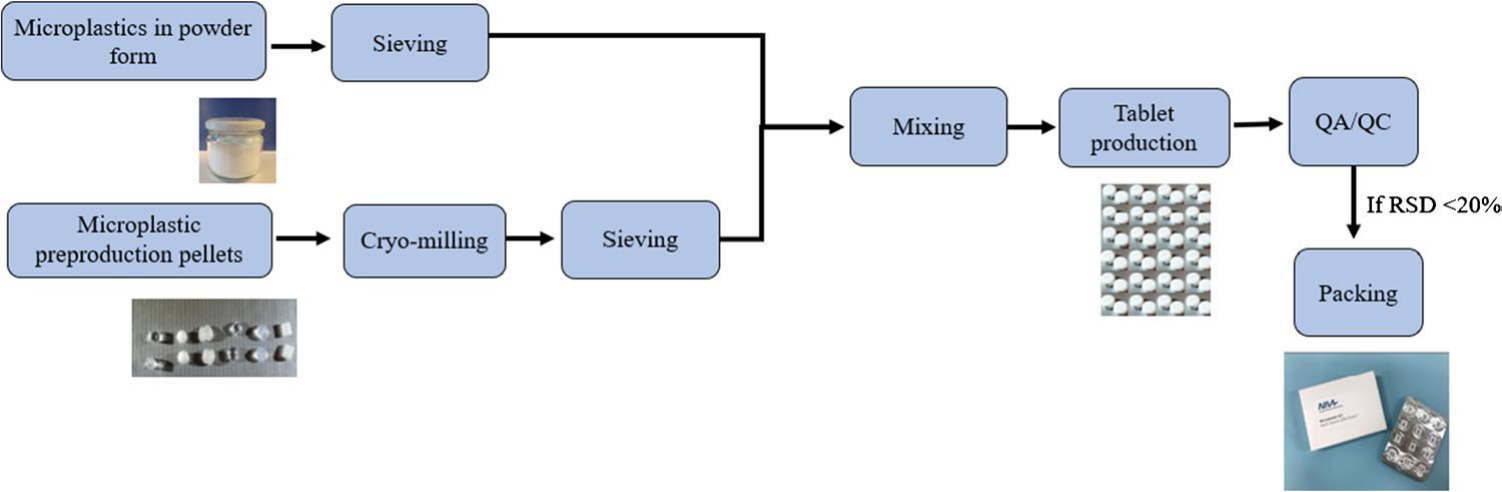
Production of candidate microplastic reference materials (RMs) at the Norwegian Institute for Water Research (NIVA, Oslo)

Soda tablets were produced using a mixture of NaHCO_3_ (Kragerø Tablet Production AS, Norway), citric acid (C_6_H_8_O_7_, VWR, Norway), and lactose (C_12_H_22_O_11_, Sigma-Aldrich, Norway). An example of the production of these pressed powder soda tablets is provided in Supplementary material, Fig. S1. A different formulation of tablet mixture was produced for soda tablets compared to soda capsules because a filler was needed. Lactose was used as a binder needed for manufacturing tablets and compacts. Once the mixture was prepared, microplastic particles of different size fractions in single or combinations of polymers were added and mixed thoroughly using a shaker (300 rpm; 1 h). Tablets could be produced containing an approximate number of plastic particles based on an initial dosing by mass, relative to the polymer density and particle size. Tablets were packed in medical tablet strips to facilitate easy shipping and storage (Fig. 2). At the beginning of the tablet production, the mixture was pressed into shape using a tablet mold with the help of a hammer. Later, a tablet press (Foshan Nanhai Shanghang Technology Limited, Guangdong, China) was purchased to assist in tablet production. This reduced tablet preparation time, providing more reproducible results.

Soda tablets containing PET fibers were made manually. PET fibers were individually isolated under a microscope and were encapsulated into a small amount of soda tablet mixture (NaHCO_3_, C_6_H_8_O_7_, C_12_H_22_O_11_) that was formed into a dough with the addition of few drops of methanol. This allowed the mixture to be wetted without the addition of water, which would have started the effervescent reaction. These small amounts of dough were then manually combined with the “bulk” soda into a tablet mold before pressing.

### Contamination control

Microplastic and blank RMs were prepared in a semi-clean room facility at the Norwegian Institute for Water Research (NIVA) microplastic laboratory, which has a positive pressure room with HEPA-filtered (class H13) air input. Several contamination reduction procedures were in place. For instance, the use of natural fiber scrubs worn under 100% cotton laboratory coats and the removal of loose fibers using a lint roller before entering the laboratory. Prior to production, all surfaces and equipment were cleaned with a 70% (filtered) ethanol solution. All laboratory RO water was pre-filtered at 0.22 µm. All containers were rinsed with filtered RO water. Entrance to this room was restricted and background contamination monitored by setting a wet filter on an open petri dish during the production. If background contamination was higher than established to be normal during routine monitoring, tablet production was stopped, the batch or batches produced on that day disposed, and the laboratory was cleaned before resuming production. In addition, blank capsules and tablets were produced alongside each batch for the different studies. This allowed tracking of potential procedural contamination during production. Results of blanks are presented in Supplementary material, Table S1 and S5-S8.

### Application of microplastic RMs in ILC studies and in-house recovery tests

#### Microplastic RMs in the form of soda capsules

Microplastic RMs in the form of soda capsules were the first type of RMs developed at NIVA. These were produced for an international ILC study coordinated by the Southern California Coastal Water Research Project Authority (SCCWRP). The aim of this study was to evaluate the performance of widely used analytical methods for microplastic analysis in drinking water, such as sample extraction, optical microscopy, FTIR, spectroscopy, and Raman. Twenty-two laboratories from six different countries participated in this study. For more information, readers are referred to the following publications [19, 20]. Three samples of simulated clean water spiked with soda capsules — containing microplastic RMs and laboratory blanks — were sent to all participants with a prescribed standard operating procedure for particle extraction, quantification, and characterization. RMs were produced to contain known amounts of microplastics within four size fractions 3–20 µm, 50–100 µm, 100–300 µm, and 300–1000 µm of the following polymers: PET, PVC, PE, and PS. The production of capsules containing the smallest size fraction (3–20 µm) was experimental due to limitations in instrument optimization on validation in the analysis of RMs in the size fraction < 50 µm. The results of QA/QC for RMs in size fractions > 50 µm are presented in the results in the “Validation and QA/QC in the production of soda capsules” section. Results of the ILC have been published in De Frond, Hampton [20].

#### Microplastic RMs in the form of soda tablets

Microplastic RMs in the form of soda tablets were produced for use in several international ILC studies as well as in-house applications for recovery tests at NIVA. Briefly, the international ILC studies in question were as follows:

1. *WEPAL-QUASIMEME/NORMAN* is a worldwide and long-term ILC exercise that was set up to assess and promote harmonization between laboratory results when analyzing microplastics [21]. In the first round of this study (2019), thirty-four institutes from sixteen countries participated in the identification and quantification of different polymer types and/or mass of particles included in the tablets. For this exercise, six preproduction pellets, PC, PS, PP, PET, LDPE, and EPS, with size fractions ranging from 2000 to 4000 μm, and six soda tablets containing microplastic were produced. One soda tablet was a blank; three contained single polymer types and size fractions, i.e., PET (150–250 µm), PVC (250–355 µm), and PS (250–355 µm); another tablet was a tri-polymer mix (of the aforementioned polymers and size fractions); and the last one contained PET fibers (101–2194 μm). Polymer quantification and identification were carried out by either counting the total number of particles in each tablet or determining the mass of particles using different analytical methods and techniques such as microscopy [22], mass spectrometry [23, 24], FTIR [25], and Raman spectroscopy [26]. Results of the ILC study were published by Van Mourik, Crum [21].
2. *INOPOL/SINOPLAST* are two international collaborative projects led by NIVA that address capacity building to reduce plastic pollution. One aim of the projects is to harmonize and improve analytical methodologies used for quantifying microplastics in India (INOPOL) and China (SINOPLAST). The objective of the ILC across these two projects was to identify polymer types of plastic pellets in the size ranges between 3 and 5 mm, as well as analyzing soda tablets containing microplastic RMs in the form of single polymer types: PE (125–150 µm), PS (250–355 µm), and PVC (150–250 µm). Seven laboratories participated in this ILC, and the analyses were conducted using different techniques such as microscopy, FTIR, Raman spectroscopy, laser direct infrared (LDIR) imaging, and pyr-GCMS. The goal of this ILC was to support ongoing training in microplastic analysis and identify the root causes of any problems affecting the quality of results.
3. *EUROqCHARM* is an ongoing EU H2020 project focused on developing, optimizing, validating, and harmonizing methods for the monitoring and assessment of plastics in the environment, as well as blueprints for standards and recommendations for policy and legislation. Together with WEPAL-QUASIMEME and the NORMAN network, an ILC study for the quantification and identification of microplastics was carried out with ninety participants from different countries. Two sets of soda tablets containing microplastics produced by cryo-milling were included in this study. One set contained PE, PET, and PS (50–300 µm) and the other contained PC, PP, and PVC (50–300 µm). Microplastic RMs were sent to all participants as soda tablets as well as sediments and sand samples pre-spiked with soda tablets. Samples were spiked individually in a slurry of sand or sediment under controlled mixing conditions in glass bottles covered with aluminum foil. All samples were sent to participants to be analyzed using their chosen analytical technique.

#### In-house application for recovery tests

NIVA routinely carries out microplastic analyses for various projects or clients. A method to validate modifications in analytical methods and ensure reproducibility of microplastic extraction and quantification from different environmental matrices was needed. Tablets produced at NIVA were used for several in-house QA/QC recovery tests. Here, QA/QC results for tablets produced and used in two projects are presented: NANOCARRIERS, which studied the effects related to micro- and nanoplastics released through wastewater treatment plants; and MIKRONOR, which has the goal to provide information on the level and type of microplastic pollution in different geographical areas in Norway. For this purpose, soda tablets containing a mixture of PE, PS, and PVC (125–355 µm) were spiked into three matrices: water, sediments, and biota.

For each recovery, tablets were dissolved into the different matrices under controlled conditions in glass beakers, covered with aluminum foil. For water, after tablets were completely dissolved, they were sieved through a 90-µm mesh and filtered through GF/A filters. Tablets containing microplastic RMs were added to biota samples in the first step during processing, i.e., when potassium hydroxide (KOH) was added to the soft tissue samples. Thereafter, samples were treated with 10% acetic acid, sieved at 90 µm, and then filtered. The same principle was applied for sediment samples, tablets were added at the beginning of processing and mixed into the sediment samples. Density separation with sodium iodide (NaI) followed by sieving and filtration was performed. For all recoveries in all matrices, beaker walls were properly rinsed to ensure all particles were transferred onto the filters prior to analysis. Analytical techniques used for characterizing the tablets were pyr-GCMS in NANOCARRIERS, and microscopy and FTIR in MIKRONOR.

### Validation of the capsules and tablets: quality assurance and quality control (QA/QC)

All capsules and tablets, irrespective of their content, underwent full QA/QC before being used in ILC studies and recovery tests. QA/QC of microplastic RMs is crucial for the use in ILC studies and the evaluation of in-house methods. It is also important for ILC studies where the variance between the tablet is smaller than the variance between the participating laboratories.

The quality of soda tablets and capsules was examined by dissolving approximately 10–20% of the total production for each batch individually in 30 mL RO water, using 100-mL beakers (one beaker per tablet). These beakers were previously cleaned and covered with aluminum foil, then the tablets or capsules were added, and placed in an incubator at 40 °C and 100 rpm for approximately 1 h, until completely dissolved. Thereafter, filtration on a 47-mm Whatman® glass microfiber filter (GF/F) with a pore size of 0.7 µm was carried out and the number of particles on each filter was counted under a stereomicroscope (Nikon, SMZ745T). The average number of particles, standard deviation (SD), and relative standard deviation (RSD) were calculated for each batch produced in each study and test. For some ILC studies, preproduction pellets were also included for analysis and their corresponding QA/QC was examined at NIVA’s laboratory.

Three statistical tests were performed on the capsule dataset, regarding average number of particles for each polymer type and size fraction, to assess the normal distribution of the data, evaluate whether there were significant differences between the different batches and to find out where the specific differences were. For this Kolmogorov–Smirnov, one-way ANOVA and Tukey–Kramer tests, respectively, were conducted and the results for each test are presented in the Supplementary material.

#### Characterization of the particle size distribution

After QA/QC, 10% of the production of two batches in the last ILC study (EUROqCHARM) were subjected to a full-size distribution characterization. For this purpose, all particles on a filter were gathered with tweezers and pictures were taken using Infinity Analyze software. Particles were numbered and the longest and shortest sides were measured based on Feret’s diameter, which is defined as the distance between two parallel tangents on opposite sides of the image for a randomly orientated particle [27]. The number of particles in these tablets was also expressed as the total number of particles in each size fraction per sample.

## Results

### *V*alidation and QA/QC in the production of soda capsules

#### SCCWRP

**Fig. 3:**
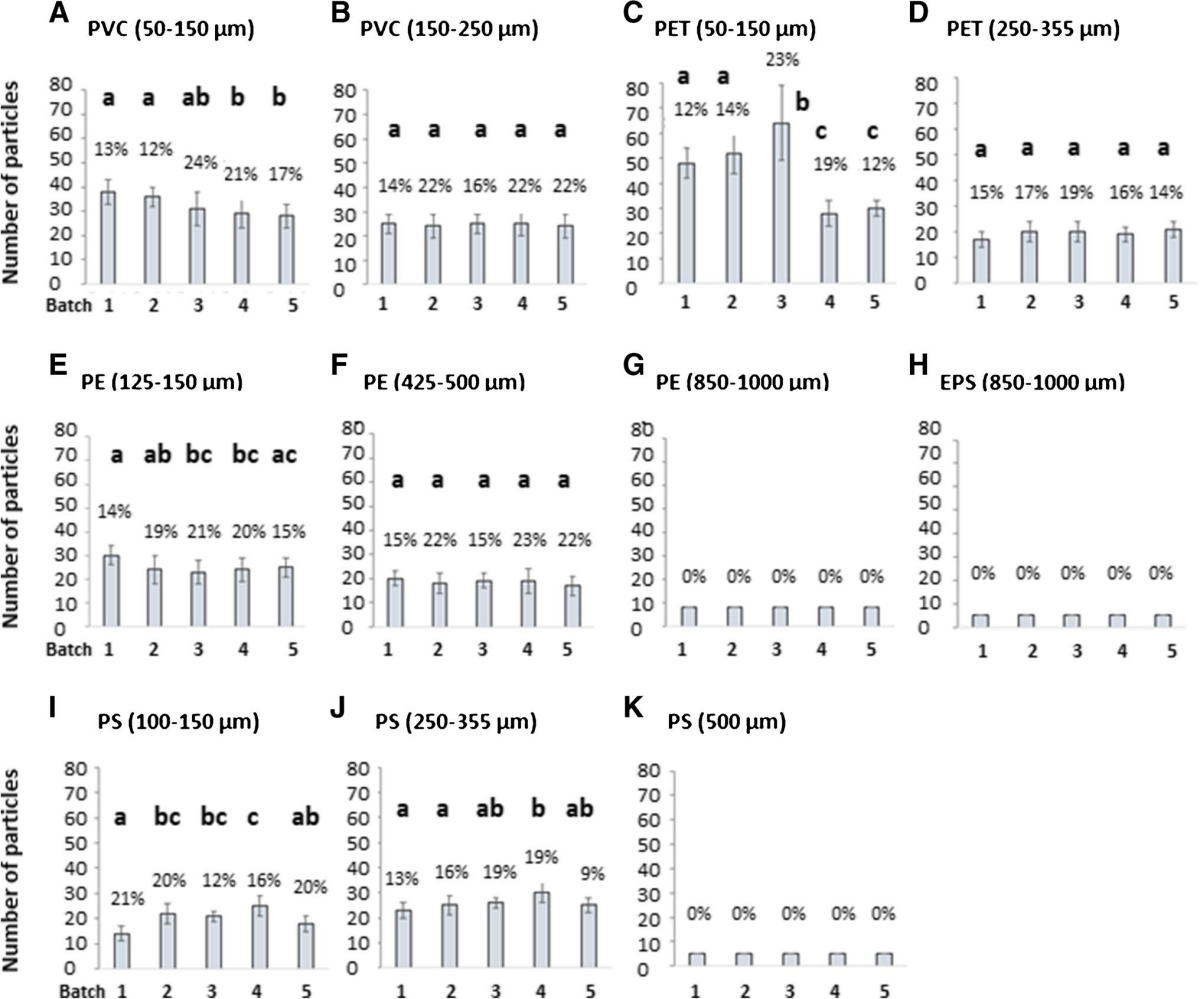
Quality assurance and quality control (QA/QC) results for the candidate microplastic reference material (RMs) produced for SCCWRP interlaboratory comparison (ILC) study as soda capsules. Graphs **A** to **K** present the number of particles and relative standard deviation (RSD) for each batch and polymer type in this ILC study. Where letters are the same on an individual graph there was no significant difference (*p* > 0.05) between these batches (one-way ANOVA with Tukey–Kramer tests)

For this study, a total of 600 capsules, divided into six sub-batches of 100 per polymer type and size fraction, were produced using a capsule maker with capacity for making 100 capsules at a time. The following polymer types and size fractions were added to each batch: PVC: 50–150 µm, 150–250 µm; PET: 50–150 µm, 250–355 µm; PE: 125–150 µm, 425–500 µm, and 850–1000 µm; EPS blue: 850–1000 µm; and PS: 100–150 µm, 250–355 µm, and 500 µm; 600 blank tablets were also produced. QA/QC for all batches was performed by dissolving 10% of the total production for each batch (“Validation of the capsules and tablets: quality assurance and quality control (QA/QC)” section), and results are presented in Fig. 3A–K. Further information on the average number of particles, SD and RSD for all batches including blanks is provided in the Supplementary material, Table S1. Two experimental batches containing PE: 3–16 µm and PS: 14–20 µm were also produced but were not subjected to QA/QC due to limitations of the analytical technique available (PerkinElmer Spotlight 400 FTIR, optimized for working from 50 µm).

**Fig. 4:**
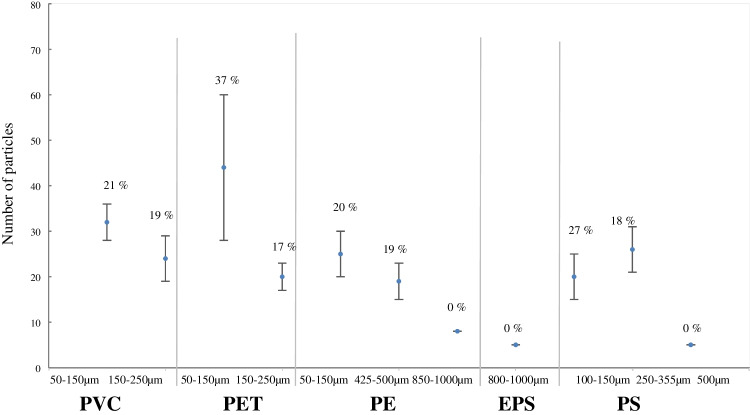
Repeatability in the production of candidate microplastic reference materials (RMs) containing single polymer types. RMs presented here were used for SCCWRP interlaboratory comparison (ILC) study

The QA/QC results for PVC in the size fractions of 50–150 µm and 150–250 µm showed a maximum RSD of 24% and 22% and a minimum RSD of 12% and 14%, respectively (Fig. 3A and B and Supplementary material, Table S1). Different batches were produced on different days using the same recipe. The repeatability of producing these batches is displayed in Fig. 4 and Supplementary material, Table S1 and presents an average number of particles of 32 ± 4 with a RSD of 21% for PVC (50–150 µm), and 24 ± 5 particles with a RSD of 19% for PVC (150–250 µm).

The results for the QA/QC for PET (50–150 µm) in batches 1 to 3 were more similar regarding the number of particles, while batches 4 and 5 presented significantly fewer particles. Maximum RSD for this polymer and size fraction was 23% and the minimum was 12%. Differences in the number of particles between batches may be explained by a possible loss of particles on the walls of the shaker during the mixing process, or by some unknown error during weighting of the polymer (Fig. 3C and Supplementary material, Table S1). Due to these differences in the number of particles between batches, the repeatability of producing PET in the fraction of 50–150 µm had an error of 37% (Fig. 4 and Supplementary material, Table S1). On the other hand, for PET in the size fraction 250–355 µm, the maximum and minimum errors in the production of the individual batches were 19% and 14%, respectively (Fig. 3D and Supplementary material, Table S1). The repeatability of the production of these batches presented an average number of particles of 20 ± 3 with a 17% RSD (Fig. 4 and Supplementary material, Table S1).

The results for PE in the three size categories: 125–150 µm, 425–500 µm, and 850–1000 µm presented a maximum RSD for their individual batches of 21%, 23%, and 0%, and a minimum RSD of 14%, 15%, and 0%, respectively (Fig. 3E, F and G and Supplementary material, Table S1). The reason for a 0% RSD in the biggest size fraction was due to the particle size, which was big enough to be put into capsules manually with low risk of particle loss. The same principle applies to the capsules containing expanded polystyrene (EPS) particles in the size fraction of 850–1000 µm (Fig. 3H and Supplementary material, Table S1). The repeatability of the three fractions for PE presented an average number of particles, SD and RSD of 25 ± 5 and 20% for the 125–150-µm size fraction, 19 ± 4 and 19% for the 425–500-µm size fraction, and 8 ± 0 and 0% for the biggest size fraction. Similarly, EPS (850–1000 µm) had an average number of particles of 5 ± 0 and a 0% RSD (Fig. 4 and Supplementary material, Table S1).

Regarding PS in the three size categories: 100–150 µm, 250–355 µm, and 500 µm, results showed a maximum RSD of 21%, 19%, and 0% and a minimum RSD of 12%, 9%, and 0%, respectively. Similar to other polymers, there was no error for the larger fraction due to their manual addition to the capsules (Fig. 3K and Supplementary material, Table S1). The repeatability for the three fractions of PS presented an average number of particles, SD and a RSD of 20 ± 5 and 27% for the 100–150 µm size fraction, 26 ± 5 and 18% for the 250–355 µm size fraction, and 5 ± 0 and 0% for the 500 µm size fraction (Fig. 4 and Supplementary material, Table S1).

The Kolmogorov–Smirnov test verified that all data, with respect to the average number of particles in all the batches, were normally distributed (*p* > 0.001). When capsule production was assessed, for single polymers, significant differences (*p* < 0.05) were identified by ANOVA between batches for some polymers. Differences were seen for PVC (50–150 µm), PET (50–150 µm), PE (125–150 µm), PS (100–150 µm), and PS (250–355 µm) (Fig. 3). The smaller the particles in the tablets, the more variation was seen between batches. There were no significant differences for the larger particles because they were individually spiked: PE (850–1000 µm), EPS (850–1000 µm), and PS (500 µm) (Fig. 3). This is also true for the larger fractions of PVC, PET, and PE. However, there were significant differences in both lower size ranges of PS. Considering there were no significance differences in batches for the larger size, this is likely a consequence of working with smaller particles. As a result, this highlights that when using this method of capsule spiking, dosing should be batch dependent and the RSDs consulted.

### Validation and QA/QC in the production of soda tablets in ILC studies

#### WEPAL/QUASIMEME/NORMAN

All participants in this ILC study received a twelve-spot blister pack with six preproduction pellets and six soda tablets. QA/QC for the soda tablets (“Validation of the capsules and tablets: quality assurance and quality control (QA/QC)” section) was calculated by dissolving 10% of the total production for each polymer type and size fraction and counting the total number of particles in the single polymer tablets and polymer mixture under a microscope.

**Fig. 5:**
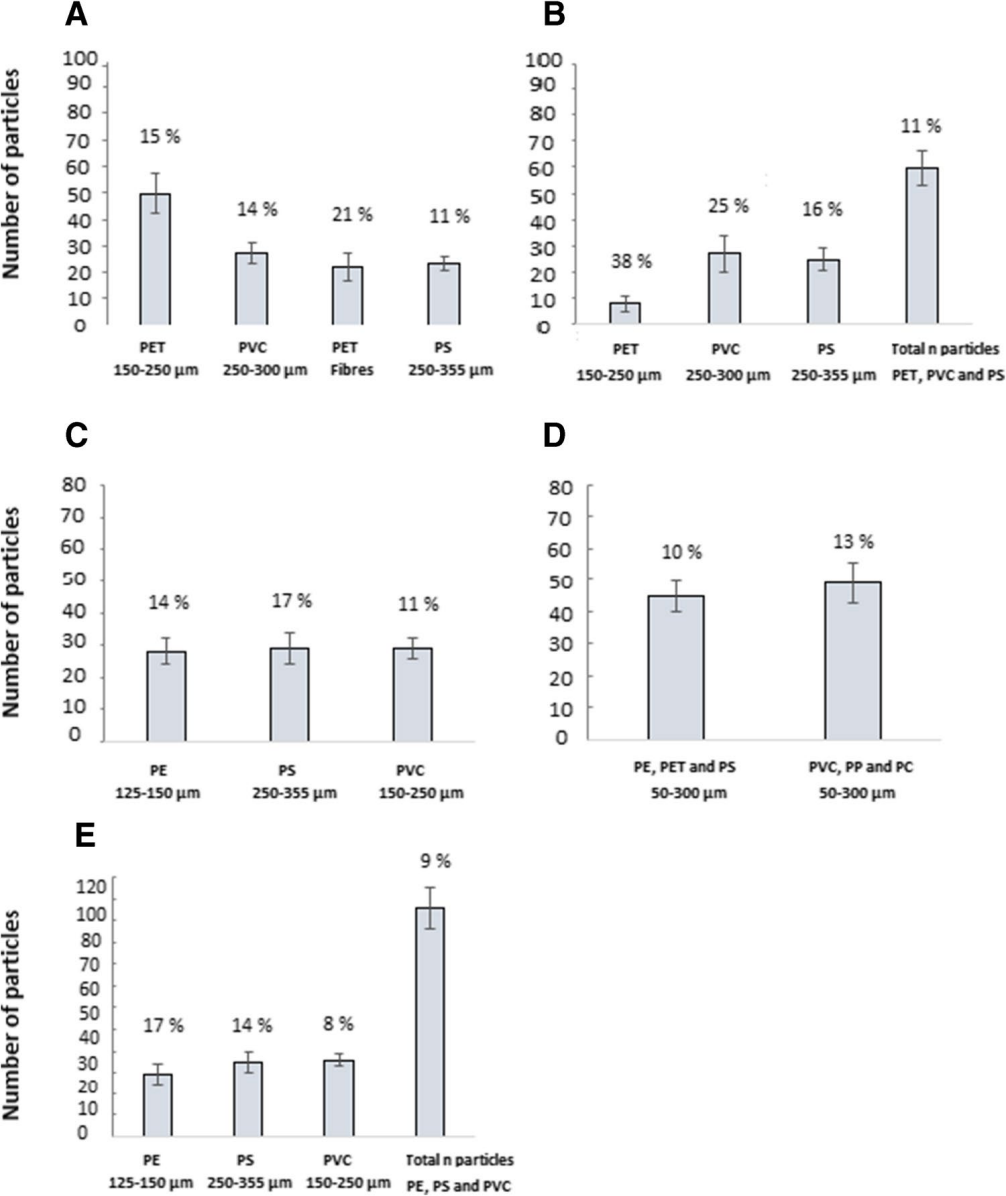
Quality assurance and quality control (QA/QC) results of interlaboratory comparison (ILC) studies using candidate microplastic reference materials (RMs) as soda tablets. Specifically, RMs used in WEPAL/QUASIMEME/NORMAN ILC study are presented for single polymers (**A**) and tablets with a mixture for 3 polymer types (**B**). Results for the soda tablets used for INOPOL/SINOPLAST ILC study containing single polymers 125–355 µm (**C**), and RMs for EUROqCHARM /QUASIMEME/NORMAN ILC study containing a mixture of 3 polymer types: PET, PET, and PS and PVC, PP, and PC in the size fractions from 50 to 300 µm (**D**). Polymer mixtures used at the Norwegian Institute for Water Research (NIVA) for in-house recovery test (**E**)

The results for the single polymer soda tablets presented a maximum RSD of 21% for PET fibers, and a minimum RSD of 11% for PS (250–355 μm), with several particles with a number varying from 50 to 22 (Fig. 5A). QA/QC for the mixed soda tablet had a better RSD for the total particle number compared to when separated for each individual polymer (Fig. 5B). This can be explained by the fact that a smaller number of particles will always present a higher RSD than a bigger number of particles. Results, including blank tablets (± 3 particles), are presented in the Supplementary material, Table S5.

#### INOPOL/SINOPLAST

QA/QC results for the three batches produced for the INOPOL/SINOPLAST ILC study presented an average number of particles, SD and RSD of 28 ± 4 and 14%, 29 ± 5 and 17%, and 29 ± 3 and 11%, for PET (125–150 µm), PS (250–355 µm), and PVC (150–250 µm), respectively (Fig. 5C). Results, including blank tablets (± 2 fibers), are presented in the Supplementary material file, Table S6.

#### EUROqCHARM /QUASIMEME/NORMAN

QA/QC performed for the two RM batches produced for the EUROqCHARM ILC study contained PE, PET, and PS and PVC, PP, and PC in size fractions between 50 and 300 µm showed an average number of particles of 45 ± 5 with a 10% RSD and 49 ± 6 particles with a 13% RSD, respectively (Fig. 5D). Results, including the blanks (containing an average of ± 2 fibers), are shown in the Supplementary material, Table S7.

Particle size distribution in ten tablets of each batch, i.e., PE, PET, and PS and PVC, PP, and PC, is presented in the Supplementary materials, Figures S2 and S3. Histograms are also presented in the Supplementary material, Figure S4 and S5.

### Validation and QA/QC for in-house recovery test in NANOCARRIERS and MIKRONOR

NIVA has used two different sets of soda tablets for in-house recovery tests, one containing PE, PET, and PS (50–300 µm), and the other containing PE (125–150 µm), PS (250–355 µm), and PVC (150–250 µm). Results for the first and second sets of soda tablets presented an average number of particles, SD and RSD of 45 ± 5 particles and 10% for the first set containing PE, PET, and PS (50–300 µm), and 29 ± 5 particles and 17%, 35 ± 5 particles and 14%, and 36 ± 3 particles and 8% for PE (125–150 µm), PS (250–355 µm), and PVC (150–250 µm), respectively, in the second set of soda tablets (Fig. 5D and E). Results for the blanks of each batch (± 2 particles, ± 2 fibers) are shown in the Supplementary material, Table S7 and S8.

## Discussion

The production of microplastic RMs provides a new approach for easy-to-use RMs as single or mixtures of different polymer types in size fractions from 50 µm to 1 mm. It is crucial to validate analytical methods and reach harmonization between laboratories while performing microplastic analyses. Microplastic RMs produced in this study were used in several ILC studies worldwide with many laboratories participating. Specific results of the ILC studies are reported elsewhere and showed that the variation between laboratories was larger than the variation between capsules and tablets.

In this study, all batches of microplastic RMs, containing single and mixtures of polymer types and size fractions, were QA/QC by NIVA’s microplastic laboratory. Results for single polymer soda capsules and tablets showed RSDs varying from 0% (for the larger particles added manually) to 24% and from 8 to 21%, respectively, for single batches. The repeatability of soda capsules showed a variation from 0 to 37% depending on the size. Moreover, RSD values varied from 8 to 38% for the multiple polymer soda tablets when individual polymers in a combined tablet were counted, and below 11% when all polymers were counted as total number of particles. Although the manual approach for making tablets containing fibers was successful, it is not feasible to make large amounts of tablets with fibers because it is time consuming. Therefore, the method presented here requires further modification and optimization to address more challenging particle types, such as microplastic fibers. Furthermore, the highest RSDs were seen in the beginning of capsule and tablet production, and subsequently improved and reduced the variation in the reference materials. Capsule production was discontinued and replaced by tablets. The driving reason behind this was that during the ILC when capsules were spiked into different matrices, the gelatin was seen to not dissolve completely when using some analytical methods [20]. On the other hand, the analytical techniques were not hindered by soda tablets. Based on the overall QA/QC presented here and continued improvements, microplastic RMs in the form of soda tablets will be suited for certification in the near future, as a tool to support and validate analytical methods and protocols.

Other attempts for microplastic RMs production include Seghers, Stefaniak [2] who made a PET batch from 30 to 200 µm by cryo-milling and wet sieving to obtain a suspension containing 800 particles/mL. NaCl was then added to obtain a salt solution, and aliquots of 1 mL of NaCl/PET were transferred to 10-mL amber glass vials. QA/QC for this batch was carried out by two different laboratories and RSDs of 16.4% and 17.9% were obtained. This is in concordance with our QA/QC results, where we consider as acceptable a RSD value < 20%. Von der Esch, Lanzinger [18] also produced microplastic RMs with a rapid sonication-based fragmentation method for PS, PET, and polylactic acid (PLA) to produce high-purity microplastic reference particles in aqueous media. Fragments were produced in the range from 100 nm to 1 mm, yielding up to 10^5^ per 15 mL microplastic particles in aqueous media. RSD values regarding the total number of particles are not reported and thus cannot be compared to our QA/QC results. In another study, Matsueda, M. [28] prepared a mixture of eleven common polymers: PE, PP, PVC, PC, PS, PET, polymethyl methacrylate, acrylonitrile butadiene styrene, nylon 6, nylon 66, and polyurethane that could be used as reference sample for microplastics analysis in environmental samples by pyr-GCMS. Results showed that the proposed mixture could be used in pyr-GCMS analyses of microplastics as a reliable reference material for at least nine of the eleven investigated polymers. It is not possible, however, to compare to our study because these results are reported in mass.

Soda tablets with MPs > 50 µm were made with sufficient precision to be used successfully both for internal recovery tests and for external ILC studies. However, for smaller MPs (< 50 µm), the variation was still too large to be used as a QC/QC tool at present. It has been shown that the number of particles in the lower size range increases exponentially in some environmental samples. It is therefore important that the concept is expanded to smaller microplastics in the future.

## Conclusion

Easy-to-use microplastic RMs (> 50 µm) using soda tablets were successfully demonstrated for several ILC studies and internal evaluation of microplastic recovery tests related to different sample preparation methods. The use of medical gelatin capsules was also tested, and although capsule production requires less resources, and showed good repeatability, the use of gelatin caused some practical problems especially in the lower size range and more specifically in the final analysis. The concept of using soda tablets worked well for several polymers: PP, PE, PS, PET, PVC, and PC, both individually and as a mixture. This was achieved for small and large quantities of MPs; RSDs were satisfactory for both individual polymers and mixtures. Reference materials are a vital tool for validation and harmonization of analytical methods for microplastic analysis and further development into the smaller MP range < 50 µm and nano range using soda tablets is urgently needed.

## Supplementary Information

Below is the link to the electronic supplementary material.

Supplementary file1 (DOCX 2.52 MB)
