# Supplementary material for: Innovative reference materials for method validation in microplastic analysis including interlaboratory comparison exercises
Source: Anal Bioanal Chem. 2023 Mar 22;415(15):2907–19. doi: 10.1007/s00216-023-04636-4 (PMC10285000; doi:10.1007/s00216-023-04636-4)
Supplement: Supplementary file 1 — Supplementary file1 (DOCX 2.52 MB) [file 216_2023_4636_MOESM1_ESM.docx]

Supplementary Material

Innovative Reference Materials for Methods Validation in Microplastic Analysis Including Interlaboratory Comparison Exercises.

Elena Martínez-Francés^1^, Bert van Bavel^1^, Rachel Hurley^1^, Luca Nizzetto^1,2^, Svetlana Pakhomova^1^, Nina T. Buenaventura^1^, Cecilie Singdahl-Larsen^1^, Marie-Louise Tambo Magni^3^, Jon Eigill Johansen^3^, Amy Lusher^1^.

^1^ Norwegian Institute for Water Research (NIVA), Økernveien 94, NO-0579 Oslo, Norway

^2^ RECETOX, Masarik University, Kamenice 753/5, 625 00 Brno, Czech Republic

^3^ Chiron AS, Stiklestadveien 1, 7041, Trondheim, Norway


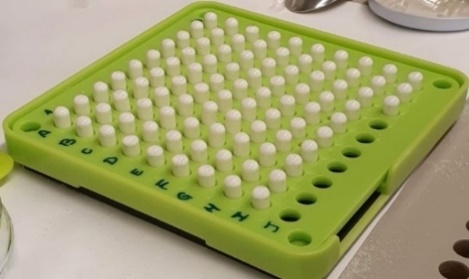

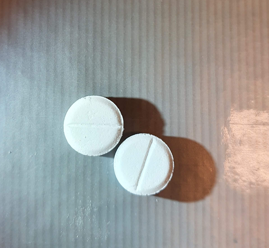


Figure S1 Example of the production of a batch for the candidate microplastic reference materials (RMs) in the form of soda capsules (left) and the production of candidate microplastic RMs in the form of soda tablets (right).

**Table S1** QA/QC results for the candidate microplastic reference materials (RMs) produced as soda capsules and used in SCCWRP interlaboratory comparison (ILC) study.

| **Polymer type and size** | **Batch 1**  (n ± SD; RSD) | **Batch 2**  (n ± SD; RSD) | **Batch 3**  (n ± SD; RSD) | **Batch 4**  (n ± SD; RSD) | **Batch 5**  (n ± SD; RSD) | **Repeatability**  (n ± SD; RSD) |
| --- | --- | --- | --- | --- | --- | --- |
| PVC (50-150 µm) | 38 ± 5; 13% | 36 ± 4; 12% | 31 ± 7; 24% | 29 ± 6; 21% | 28 ± 5; 17% | 32 ± 4; 21% |
| PVC (150-250 µm) | 25 ± 4; 14% | 24 ± 5; 22% | 25 ± 4; 16% | 25 ± 5; 22% | 24 ± 5; 22% | 24 ± 5; 19% |
| PET (50-150 µm) | 48 ± 6; 12% | 52± 8; 14% | 64 ± 15; 23% | 28 ± 5; 19% | 30 ± 4; 12% | 44 ± 16; 37% |
| PET (250-355 µm) | 17 ± 3; 15% | 20 ± 4; 17% | 20 ± 4; 19% | 19 ± 3; 16% | 21 ± 3; 14% | 20 ± 3; 17% |
| PE (125-150 µm) | 30 ± 4; 14% | 25 ± 5; 19% | 23 ± 5; 21% | 24 ± 5; 20% | 25 ± 4; 15% | 25 ± 5; 20% |
| PE (425-500 µm) | 20 ± 3; 15% | 18 ± 4; 22% | 19 ± 3; 15% | 19 ± 5; 23% | 17 ± 4; 22% | 19 ± 4; 19% |
| PE (850-1000 µm) | 8 ± 0% | 8 ± 0% | 8 ± 0% | 8 ± 0% | 8 ± 0% | 8; 0% |
| EPS blue (800-1000 µm) | 5 ± 0% | 5 ± 0% | 5 ± 0% | 5 ± 0% | 5 ± 0% | 5; 0% |
| PS (100-150 µm) | 14 ± 3; 21% | 22 ± 4; 20% | 21 ± 2; 12% | 25 ± 4; 16% | 18 ± 3; 20% | 20 ± 5; 27% |
| PS (250-355 µm) | 23 ± 3; 13% | 25 ± 4; 16% | 26 ± 5; 19% | 30 ± 6; 19% | 25 ± 2; 9% | 26 ± 5; 18% |
| PS (500 µm) | 5 ± 0% | 5 ± 0% | 5 ± 0% | 5 ± 0% | 5 ± 0% | 5 ± 0% |
| Blanks | 0 particles 1 fiber | 0 particles 2 fibers | 1 particle 0 fiber | 0 particles 2 fibers | 0 particles 3 fiber | ± 0 particle ± 2 fibers |

**Table S2** Results for Kolmogorov-Smirnov test to study the normal distribution for the dataset of all the batches, regarding average number of particles, for each polymer type and size fraction produced for SCCWRP interlaboratory comparison (ILC) study.

|  | **PVC**  **(50-150 µm)** | **PVC**  **(150-250 µm)** | **PET**  **(50-150 µm)** | **PET**  **(250-355 µm)** | **PE**  **(125-150 µm)** | **PE**  **(425-500 µm)** | **PS**  **(100-150 µm)** | **PS**  **(100-150 µm)** |
| --- | --- | --- | --- | --- | --- | --- | --- | --- |
| **Count** | 50.00 | 50.00 | 49.00 | 50.00 | 50.00 | 50.00 | 51.00 | 49.00 |
| **Mean** | 32.42 | 24.40 | 44.22 | 19.66 | 25.36 | 18.80 | 19.71 | 25.78 |
| **Median** | 33.00 | 25.00 | 43.00 | 19.00 | 25.00 | 19.00 | 20.00 | 25.00 |
| **SD** | 6.82 | 4.66 | 16.31 | 3.37 | 5.29 | 3.63 | 5.29 | 4.58 |
| **Skewness** | 0.28 | -0.27 | 0.59 | 0.35 | 0.20 | -0.17 | 0.01 | 0.81 |
| **Kurtosis** | -0.71 | -0.74 | -0.33 | -0.49 | -0.30 | -0.55 | -0.55 | 0.05 |
| **KS (D)** | 0.08 | 0.08 | 0.12 | 0.14 | 0.10 | 0.15 | 0.11 | 0.14 |
| **P value** | 0.92 | 0.89 | 0.40 | 0.27 | 0.70 | 0.19 | 0.49 | 0.25 |

**Table S3** Results for one-way ANOVA test to identify possible significant differences between the average number of particles for the different batches produced for SCCWRP interlaboratory comparison (ILC) study.

|  | | | | |  |
| --- | --- | --- | --- | --- | --- |
| **PVC (50-150 µm)** | | | | | |
| **Source** | **SS** | **df** | **MS** |  |  |
| Between-treatments | 824.28 | 4 | 206.07 | F=6.38 | P = 3.7510^-3^ |
| Within-treatments | 1453.90 | 45 | 32.31 |  |  |
| Total | 2278.18 | 49 |  |  |  |
| **PVC (150-250 µm)** | | | | | |
| **Source** | **SS** | **df** | **MS** |  |  |
| Between-treatments | 30.60 | 4 | 7.65 | F=0.33 | P = 0.85 |
| Within-treatments | 1031.40 | 45 | 22.92 |  |  |
| Total | 1062.00 | 49 |  |  |  |
| **PET (50-150 µm)** | | | | | |
| **Source** | **SS** | **df** | **MS** |  |  |
| Between-treatments | 9557.04 | 4 | 2389.26 | F=32.77 | P = 1.11x10^-12^ |
| Within-treatments | 3207.49 | 44 | 72.90 |  |  |
| Total | 12764.53 | 48 |  |  |  |
| **PET (250-355 µm)** | | | | | |
| **Source** | **SS** | **df** | **MS** |  |  |
| Between-treatments | 82.12 | 4 | 20.53 | F=1.94 | P = 0.12 |
| Within-treatments | 475.10 | 45 | 10.56 |  |  |
| Total | 557.22 | 49 |  |  |  |
| **PE (125-150 µm)** | | | | | |
| **Source** | **SS** | **df** | **MS** |  |  |
| Between-treatments | 370.12 | 4 | 92.53 | F=4.16 | P = 5.91 x10^-3^ |
| Within-treatments | 999.40 | 45 | 22.21 |  |  |
| Total | 1369.52 | 49 |  |  |  |
| **PE (425-500 µm)** | | | | | |
| **Source** | **SS** | **df** | **MS** |  |  |
| Between-treatments | 36.40 | 4 | 9.10 | F=0.67 | P = 0.61 |
| Within-treatments | 607.60 | 45 | 13.50 |  |  |
| Total | 644.00 | 49 |  |  |  |
| **PS (100-150 µm)** | | | | | |
| **Source** | **SS** | **df** | **MS** |  |  |
| Between-treatments | 808.66 | 4 | 202.16 | F=15.76 | P = 3.4x10^-8^ |
| Within-treatments | 589.93 | 46 | 12.82 |  |  |
| Total | 1398.59 | 50 |  |  |  |
| **PS (250-355 µm)** | | | | | |
| **Source** | **SS** | **df** | **MS** |  |  |
| Between-treatments | 248.51 | 4 | 62.13 | F=3.60 | P =1.26 x10^-2^ |
| Within-treatments | 758.02 | 44 | 17.23 |  |  |
| Total | 1006.53 | 48 |  |  |  |
|  |  |  |  |  |  |

**Table S4** Results for Tukey-Kramer test run after one-way ANOVA test to assess where the differences, regarding the average number of particles, between batches produced for SCCWRP interlaboratory comparison (ILC) study were.

| **Pairwise Comparisons PVC (50-150 µm)** | | **HSD_.05_ = 7.22** | **Q_.05_ = 4.02** | **Pairwise Comparisons PE (125-150 µm)** | | **HSD_.05_ = 5.99** | **Q_.05_ = 4.02** |
| --- | --- | --- | --- | --- | --- | --- | --- |
|  |  | **HSD_.01_ = 8.79** | **Q_.01_ = 4.89** |  |  | **HSD_.01_ = 7.29** | **Q_.01_ = 4.89** |
| **T_1_:T_2_** | M_1_ = 37.90 | 1.4 | Q = 0.78 (p = 0.98) | **T_1_:T_2_** | M_1_ = 30.50 | 5.9 | Q = 3.96 (p = 0.06) |
|  | M_2_ = 36.50 |  |  |  | M_2_ = 24.60 |  |  |
| **T_1_:T_3_** | M_1_ = 37.90 | 6.9 | Q = 3.84 (p = 0.07) | **T_1_:T_3_** | M_1_ = 30.50 | 7.0 | Q = 4.70 (p = 0.02) |
|  | M_3_ = 31.00 |  |  |  | M_3_ = 23.50 |  |  |
| **T_1_:T_4_** | M_1_ = 37.90 | 9.0 | Q = 5.01 (p = 0.01) | **T_1_:T_4_** | M_1_ = 30.50 | 7.7 | Q = 5.17 (p = 6.00 x10^-2^) |
|  | M_4_ = 28.90 |  |  |  | M_4_ = 22.80 |  |  |
| **T_1_:T_5_** | M_1_ = 37.90 | 10.1 | Q = 5.62 (p = 2.00 x10^-2^) | **T_1_:T_5_** | M_1_ = 30.50 | 5.1 | Q = 3.42 (p = 0.13) |
|  | M_5_ = 27.80 |  |  |  | M_5_ = 25.40 |  |  |
| **T_2_:T_3_** | M_2_ = 36.50 | 5.5 | Q = 3.06 (p = 0.21) | **T_2_:T_3_** | M_2_ = 24.60 | 1.1 | Q = 0.74 (p = 0.98) |
|  | M_3_ = 31.00 |  |  |  | M_3_ = 23.50 |  |  |
| **T_2_:T_4_** | M_2_ = 36.50 | 7.6 | Q = 4.23 (p = 0.04) | **T_2_:T_4_** | M_2_ = 24.60 | 1.8 | Q = 1.21 (p = 0.91) |
|  | M_4_ = 28.90 |  |  |  | M_4_ = 22.80 |  |  |
| **T_2_:T_5_** | M_2_ = 36.50 | 8.7 | Q = 4.84 (p = 0.01) | **T_2_:T_5_** | M_2_ = 24.60 | 0.8 | Q = 0.54 (p = 0.99) |
|  | M_5_ = 27.80 |  |  |  | M_5_ = 25.40 |  |  |
| **T_3_:T_4_** | M_3_ = 31.00 | 2.1 | Q = 1.17 (p = 0.92) | **T_3_:T_4_** | M_3_ = 23.50 | 0.7 | Q = 0.47 (p = 1.00) |
|  | M_4_ = 28.90 |  |  |  | M_4_ = 22.80 |  |  |
| **T_3_:T_5_** | M_3_ = 31.00 | 3.2 | Q = 1.78 (p = 0.72) | **T_3_:T_5_** | M_3_ = 23.50 | 1.9 | Q = 1.27 (p = 0.90) |
|  | M_5_ = 27.80 |  |  |  | M_5_ = 25.40 |  |  |
| **T_4_:T_5_** | M_4_ = 28.90 | 1.1 | Q = 0.61 (p = 0.99) | **T_4_:T_5_** | M_4_ = 22.80 | 2.6 | Q = 1.74 (p = 0.73) |
|  | M_5_ = 27.80 |  |  |  | M_5_ = 25.40 |  |  |
| **Pairwise Comparisons PVC (150-250 µm)** | | **HSD_.05_ = 6.08** | **Q_.05_ = 4.02** | **Pairwise Comparisons PE (425-500 µm)** | | **HSD_.05_ = 4.67** | **Q_.05_ = 4.02** |
|  |  | **HSD_.01_ = 7.41** | **Q._01_ = 4.89** |  |  | **HSD_.01_ = 5.68** | **Q_.01_ = 4.89** |
| **T_1_:T_2_** | M_1_ = 24.70 | 1.5 | Q = 0.99 (p = 0.95) | **T_1_:T_2_** | M_1_ = 19.90 | 1.8 | Q = 1.55 (p = 0.81) |
|  | M_2_ = 23.20 |  |  |  | M_2_ = 18.10 |  |  |
| **T_1_:T_3_** | M_1_ = 24.70 | 0.3 | Q = 0.20 (p = 1.00) | **T_1_:T_3_** | M_1_ = 19.90 | 1.0 | Q = 0.86 (p = 0.97) |
|  | M_3_ = 25.00 |  |  |  | M_3_ = 18.90 |  |  |
| **T_1_:T_4_** | M_1_ = 24.70 | 0.6 | Q = 0.40 (p = 1.00) | **T_1_:T_4_** | M_1_ = 19.90 | 0.4 | Q = 0.34 (p = 1.00) |
|  | M_4_ = 25.30 |  |  |  | M_4_ = 19.50 |  |  |
| **T_1_:T_5_** | M_1_ = 24.70 | 0.9 | Q = 0.59 (p = 0.99) | **T_1_:T_5_** | M_1_ = 19.90 | 2.3 | Q = 1.98 (p = 0.631 |
|  | M_5_ = 23.80 |  |  |  | M_5_ = 17.60 |  |  |
| **T_2_:T_3_** | M_2_ = 23.20 | 1.8 | Q = 1.19 (p = 0.92) | **T_2_:T_3_** | M_2_ = 18.10 | 0.8 | Q = 0.69 (p = 0.99) |
|  | M_3_ = 25.00 |  |  |  | M_3_ = 18.90 |  |  |
| **T_2_:T_4_** | M_2_ = 23.20 | 2.1 | Q = 1.39 (p = 0.86) | **T_2_:T_4_** | M_2_ = 18.10 | 1.4 | Q = 1.20 (p = 0.91) |
|  | M_4_ = 25.30 |  |  |  | M_4_ = 19.50 |  |  |
| **T_2_:T_5_** | M_2_ = 23.20 | 0.6 | Q = 0.40 (p = 1.00) | **T_2_:T_5_** | M_2_ = 18.10 | 0.5 | Q = 0.43 (p = 1.00) |
|  | M_5_ = 23.80 |  |  |  | M_5_ = 17.60 |  |  |
| **T_3_:T_4_** | M_3_ = 25.00 | 0.3 | Q = 0.20 (p = 1.00) | **T_3_:T_4_** | M_3_ = 18.90 | 0.6 | Q = 0.52 (p = 1.00) |
|  | M_4_ = 25.30 |  |  |  | M_4_ = 19.50 |  |  |
| **T_3_:T_5_** | M_3_ = 25.00 | 1.2 | Q = 0.79 (p = 0.98) | **T_3_:T_5_** | M_3_ = 18.90 | 1.3 | Q = 1.12 (p = 0.93) |
|  | M_5_ = 23.80 |  |  |  | M_5_ = 17.60 |  |  |
| **T_4_:T_5_** | M_4_ = 25.30 | 1.5 | Q = 0.99 (p = 0.96) | **T_4_:T_5_** | M_4_ = 19.50 | 1.9 | Q = 1.64 (p = 0.78) |
|  | M_5_ = 23.80 |  |  |  | M_5_ = 17.60 |  |  |
| **Pairwise Comparisons PET (50-150 µm)** | | **HSD_.05_ = 0.98** | **Q_.05_ = 4.02** | **Pairwise Comparisons**  **PS (100-150 µm)** | | **HSD_.05_ = 4.61** | **Q_.05_ = 4.01** |
|  |  | **HSD_.01_ = 3.38** | **Q_.01_= 4.81** |  |  | **HSD_.01_ = 5.61** | **Q._01_ = 4.89** |
| **T_1_:T_2_** | M_1_ = 47.89 | 4.41 | Q = 1.62 (p = 0.78) | **T_1_:T_2_** | M_1_ = 14.36 | 7.9 | Q = 6.92 (p= 1.20 x10^-4^) |
|  | M_2_ = 52.30 |  |  |  | M_2_ = 22.30 |  |  |
| **T_1_:T_3_** | M_1_ = 47.89 | 16.11 | Q = 5.90 (p = 1.26 x10^-3^) | **T_1_:T_3_** | M_1_ = 14.36 | 7.1 | Q = 6.16 (p = 6.80 x10^-4^) |
|  | M_3_ = 64.00 |  |  |  | M_3_ = 21.43 |  |  |
| **T_1_:T_4_** | M_1_ = 47.89 | 20.29 | Q = 7.43 (p = 4.00 x10^-5^) | **T_1_:T_4_** | M_1_ = 14.36 | 10.7 | Q = 9.35 (p = 0.00) |
|  | M_4_ = 27.60 |  |  |  | M_4_ = 25.10 |  |  |
| **T_1_:T_5_** | M_1_ = 47.89 | 18.19 | Q = 6.66 (p = 2.30 x10^-4^) | **T_1_:T_5_** | M_1_ = 14.36 | 3.6 | Q = 3.17 (p = 0.18) |
|  | M_5_ = 29.70 |  |  |  | M_5_ = 18.00 |  |  |
| **T_2_:T_3_** | M_2_ = 52.30 | 11.7 | Q = 4.29 (p = 0.032) | **T_2_:T_3_** | M_2_ = 22.30 | 0.9 | Q = 0.76 (p = 0.98) |
|  | M_3_ = 64.00 |  |  |  | M_3_ = 21.43 |  |  |
| **T_2_:T_4_** | M_2_ = 52.30 | 24.7 | Q = 9.05 (p = 0.00) | **T_2_:T_4_** | M_2_ = 22.30 | 2.8 | Q = 2.44 (p = 0.43) |
|  | M_4_ = 27.60 |  |  |  | M_4_ = 25.10 |  |  |
| **T_2_:T_5_** | M_2_ = 52.30 | 22.6 | Q = 8.28 (p =1.00 x10^-5^) | **T_2_:T_5_** | M_2_ = 22.30 | 4.3 | Q = 3.74 (p = 0.08) |
|  | M_5_ = 29.70 |  |  |  | M_5_ = 18.00 |  |  |
| **T_3_:T_4_** | M_3_ = 64.00 | 36.4 | Q = 13.33 (p = 0.00) | **T_3_:T_4_** | M_3_ = 21.43 | 3.7 | Q = 3.20 (p = 0.18) |
|  | M_4_ = 27.60 |  |  |  | M_4_ = 25.10 |  |  |
| **T_3_:T_5_** | M_3_ = 64.00 | 34.3 | Q = 12.57 (p = 0.00) | **T_3_:T_5_** | M_3_ = 21.43 | 3.4 | Q = 2.99 (p = 0.23) |
|  | M_5_ = 29.70 |  |  |  | M_5_ = 18.00 |  |  |
| **T_4_:T_5_** | M_4_ = 27.60 | 2.1 | Q = 0.77 (p = 0.98) | **T_4_:T_5_** | M_4_ = 25.10 | 7.1 | Q = 6.18 (p = 6.40 x10^-4^) |
|  | M_5_ = 29.70 |  |  |  | M_5_ = 18.00 |  |  |
| **Pairwise Comparisons PET (250-355 µm)** | | **HSD_.05_ = 4.13** | **Q_.05_ = 4.02** | **Pairwise Comparisons PS (250-355 µm)** | | **HSD_.05_ = 5.34** | **Q_.05_ = 4.02** |
|  |  | **HSD_.01_ = 5.03** | **Q_.01_ = 4.89** |  |  | **HSD_.01_ = 6.50** | **Q_.01_ = 4.90** |
| **T_1_:T_2_** | M_1_ = 17.40 | 2.9 | Q = 2.82 (p = 0.28) | **T_1_:T_2_** | M_1_ = 23.50 | 1.06 | Q = 0.80 (p = 0.98) |
|  | M_2_ = 20.30 |  |  |  | M_2_ = 24.56 |  |  |
| **T_1_:T_3_** | M_1_ = 17.40 | 2.9 | Q = 2.82 (p = 0.28) | **T_1_:T_3_** | M_1_ = 23.50 | 2.1 | Q = 1.58 (p = 0.78) |
|  | M_3_ = 20.30 |  |  |  | M_3_ = 25.60 |  |  |
| **T_1_:T_4_** | M_1_ = 17.40 | 1.8 | Q = 1.75 (p = 0.73) | **T_1_:T_4_** | M_1_ = 23.50 | 6.5 | Q = 4.90 (p = 0.01) |
|  | M_4_ = 19.20 |  |  |  | M_4_ = 30.00 |  |  |
| **T_1_:T_5_** | M_1_ = 17.40 | 3.7 | Q = 3.60 (p = 0.09) | **T_1_:T_5_** | M_1_ = 23.50 | 1.6 | Q = 1.21 (p = 0.91) |
|  | M_5_ = 21.10 |  |  |  | M_5_ = 25.10 |  |  |
| **T_2_:T_3_** | M_2_ = 20.30 | 0 | Q = 0.00 (p = 0.00) | **T_2_:T_3_** | M_2_ = 24.56 | 1.04 | Q = 0.79 (p = 0.98) |
|  | M_3_ = 20.30 |  |  |  | M_3_ = 25.60 |  |  |
| **T_2_:T_4_** | M_2_ = 20.30 | 1.1 | Q = 1.07 (p = 0.94) | **T_2_:T_4_** | M_2_ = 24.56 | 5.44 | Q = 4.10 (p = 0.04) |
|  | M_4_ = 19.20 |  |  |  | M_4_ = 30.00 |  |  |
| **T_2_:T_5_** | M_2_ = 20.30 | 0.8 | Q = 0.78 (p = 0.98) | **T_2_:T_5_** | M_2_ = 24.56 | 0.54 | Q = 0.41 (p = 1.00) |
|  | M_5_ = 21.10 |  |  |  | M_5_ = 25.10 |  |  |
| **T_3_:T_4_** | M_3_ = 20.30 | 1.1 | Q = 1.07 (p = 0.94) | **T_3_:T_4_** | M_3_ = 25.60 | 4.4 | Q = 3.32 (p = 0.15) |
|  | M_4_ = 19.20 |  |  |  | M_4_ = 30.00 |  |  |
| **T_3_:T_5_** | M_3_ = 20.30 | 0.8 | Q = 0.78 (p = 0.98) | **T_3_:T_5_** | M_3_ = 25.60 | 0.5 | Q = 0.38 (p = 1.00) |
|  | M_5_ = 21.10 |  |  |  | M_5_ = 25.10 |  |  |
| **T_4_:T_5_** | M_4_ = 19.20 | 1.9 | Q = 1.85 (p = 0.69) | **T_4_:T_5_** | M_4_ = 30.00 | 4.9 | Q = 3.69 (p = 0.09) |
|  | M_5_ = 21.10 |  |  |  | M_5_ = 25.10 |  |  |

Table S5 Contents and QA/QC results for the plastic pellets and the candidate microplastic reference materials (RMs) used as soda tablets and produced for WEPAL/QUASIMEME/NORMAN interlaboratory comparison (ILC) study.

| Sample | Type | Polymer | Size | Average number of particles, SD and RSD |
| --- | --- | --- | --- | --- |
| 1 | Preproduction pellet | Polycarbonate (PC) | 3-5 mm |  |
| 2 | Preproduction pellet | Polystyrene (PS) | 3-5 mm |  |
| 3 | Preproduction pellet | Polypropylene (PP) | 3-5 mm |  |
| 4 | Preproduction pellet | Polyethylene terephthalate (PET) | 3-5 mm |  |
| 5 | Preproduction pellet | Low-density polyethylene (LDPE) | 3-5 mm |  |
| 6 | Preproduction pellet | Expanded polystyrene (EPS) | 3-5 mm |  |
| 7 |  | PET | 150-250 µm | 50 ± 8; RSD 15% |
| 8 |  | PVC | 250-300 µm | 27 ± 4; RSD 14% |
| 9 |  | PET fibers | 101-2194 μm | 22 ± 5; RSD 21% |
| 10 |  | PET | 150-250 μm | 8.0 ± 3; RSD 38% |
|  |  | PVC | 250-300 µm | 27 ± 7; RSD 25% |
|  |  | PS | 250-300 µm | 25 ± 4; RSD 16% |
| 11 |  | PS | 250-300 µm | 24 ± 3; RSD 11% |
| 12 |  | Blank |  | ± 3 particles |

Table S6 QA/QC results for the candidate microplastic reference materials (RMs) produced as soda tablets and used in INOPOL/SINOPLAST interlaboratory comparison (ILC) study.

| Sample | Polymer type and size fraction | Average number of particles (n ± SD and RSD) |
| --- | --- | --- |
| 1 | PET (125-150 μm) | 28 ± 4; RSD 14% |
| 2 | PS (250-355 µm) | 29 ± 5; RSD 17% |
| 3 | PVC (150-250 µm) | 29 ± 3; RSD 11% |
| 4 | Blank | ± 2 fibers |

Table S7 QA/QC results for the candidate microplastic reference materials (RMs) for soda tablets in a mixture of polymers used in EUROqCHARM/QUASIMEME/NORMAN interlaboratory comparison (ILC) study.

| Sample | Polymer | Average number of particles (n ± SD and RSD) |
| --- | --- | --- |
| 1 | PE, PET and PS  (50-300 µm) | 45 ± 5; RSD 10% |
| 2 | PVC, PP and PC (50-300 µm) | 49 ± 6; RSD 13% |
| 3 | Blank | ± 2 particles |


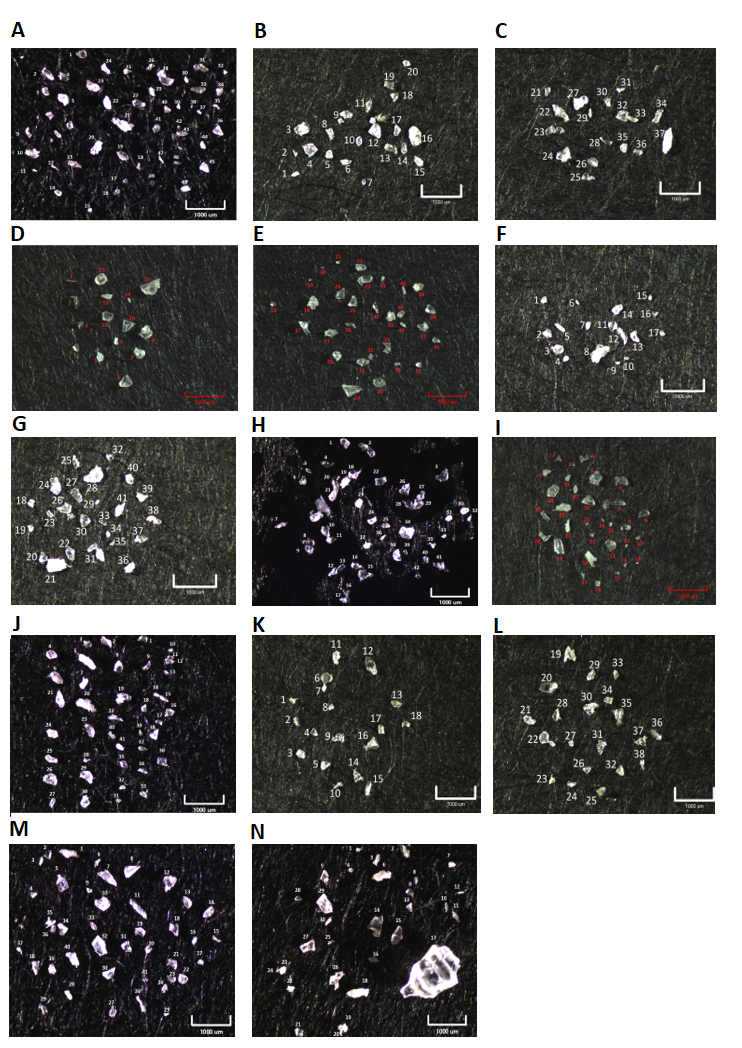


**Figure S2** QA/QC for all the particles gathered on 10 filters for EUROqCHARM /QUASIMEME/NORMAN interlaboratory comparison (ILC) study in the size range from 50-300 µm for the batch containing PET, PE and PS. Part A corresponds to filter 1, B and C to filter 2 divided in 2 parts. D and E represents filter 3. F and G filter 4. H, I and J filter 5, 6 and 7. K and L filter 8 and M and N filters 9 and 10.


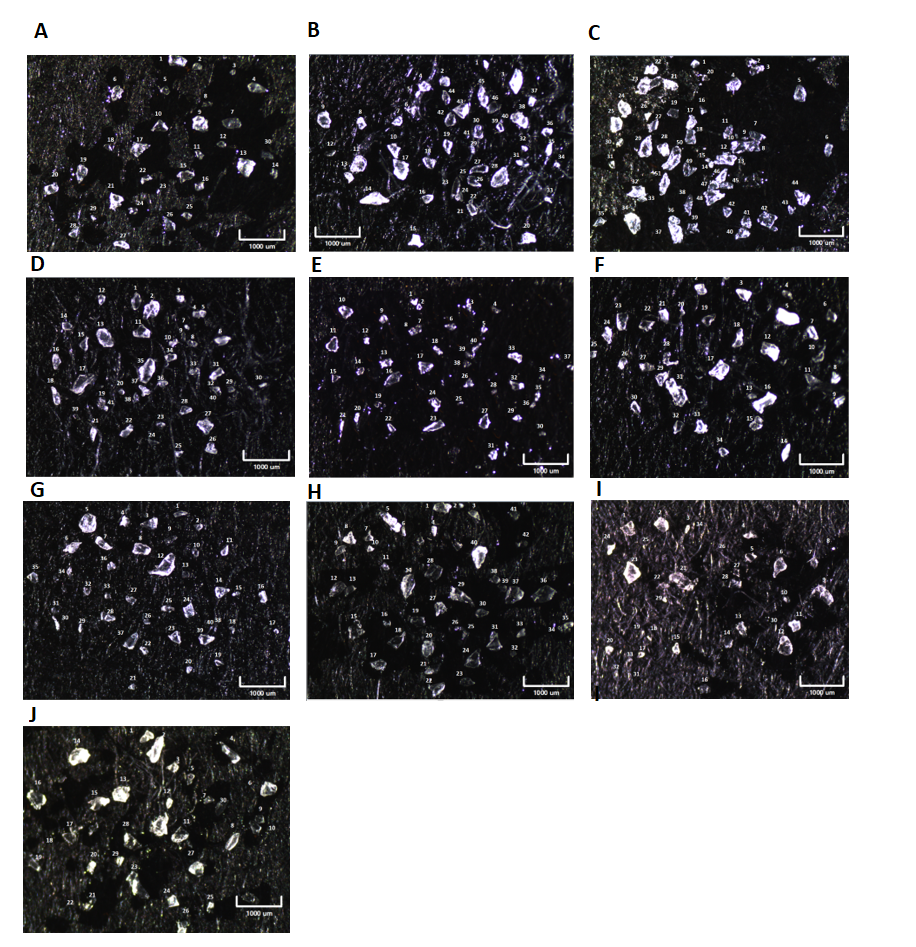


Figure S3 QA/QC for all the particles gathered on 10 filters for EUROqCHARM /QUASIMEME/NORMAN interlaboratory comparison (ILC) study in the size range from 50-300 µm for the batch containing PVC, PP and PC. Part A to J correspond to each of the filters where the particles were measured.


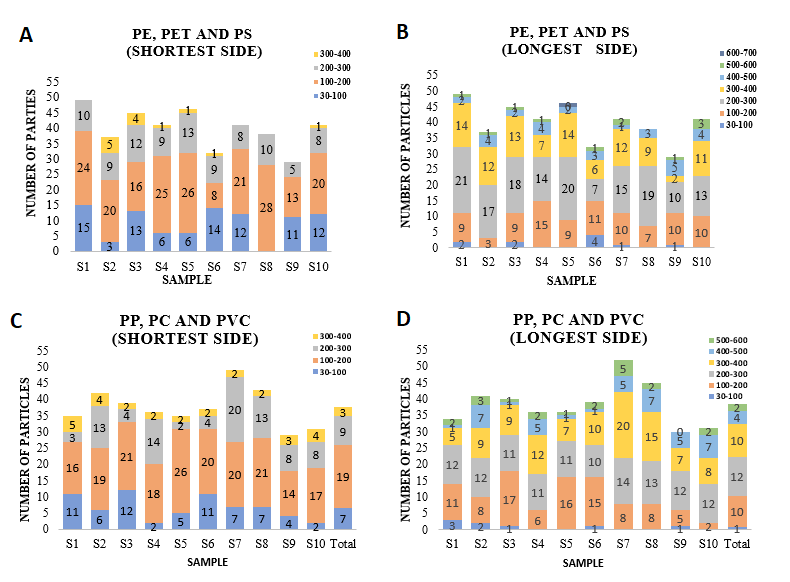


Figure S4 Particle distribution in 10 of the tablets for PE, PET and PS and PP, PVC and PP from 50-300 µm. Results are expressed as number of particles per size fraction measuring the shortest (A & C) and longest (B & D) size in each particle on each filter. for PE, PET and PS (left) and PP, PC and PVC (right).


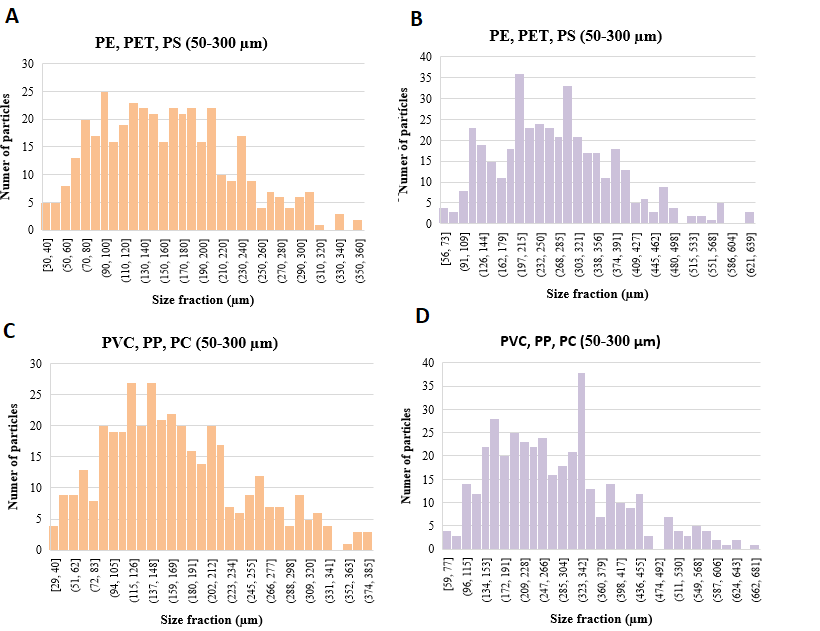


Figure S5 Histograms presenting the size distribution for PE, PET and PS and PVC, PP and PC in the size fractions from 50-300 µm. Graphs (A & C) present the shortest size fractions, and (B & D) the longest size fraction considering all the particles in 10 filters for PE, PET and PS (50- 300 µm) upper side of the figure and PP, PC and PVC (50-300 µm) lower part of figure.

Table S8 QA/QC results for the soda tablets containing a mixture of 3 polymers used as candidate microplastic reference materials (RMs) for in-house recovery tests at NIVA: Mikronor and Nanocarriers

| Sample | Polymer type | Average number of particles (n ± SD and RSD) |
| --- | --- | --- |
| 1 | PE (125-150 µm) | 29 ± 5; 17% |
|  | PS (250-355 µm) | 35 ± 5; 14% |
|  | PVC (125-250 µm) | 36 ± 3, 8% |
|  | Total number of particles | 106 ± 5, 9% |
| Blanks |  | ± 2 fibers |
